# Supplementary material for: Coding and noncoding landscape of extracellular RNA released by human glioma stem cells
Source: Nat Commun. 2017 Oct 26;8:1145. doi: 10.1038/s41467-017-01196-x (PMC5658400; doi:10.1038/s41467-017-01196-x)
Supplement: Supplementary file 1 — Supplementary Information [file 41467_2017_1196_MOESM1_ESM.pdf]

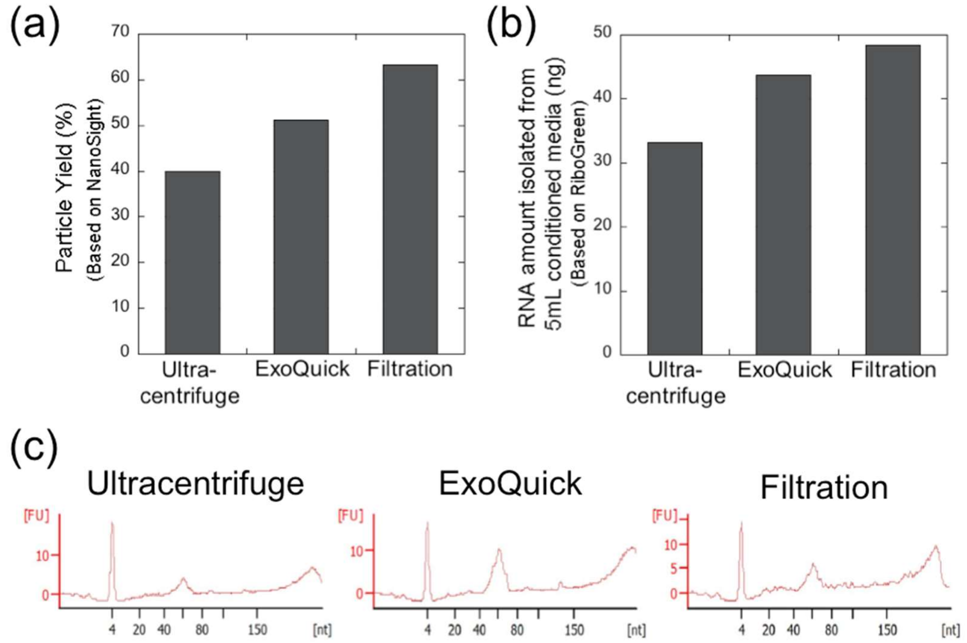

Supplementary Figure 1. Comparison of conventional ultracentrifugation at 100,000g, ExoQuick, and one-step filtration protocols for EV isolation and RNA preparation. The filtration-based protocol exhibits higher yields of particle **(a)** and RNA **(b)** isolation. The RNA profiles and quality are similar for different isolation approaches **(c)**. Of note, the one-step filtration protocol is the prototype of the presented sequential filtration protocol.

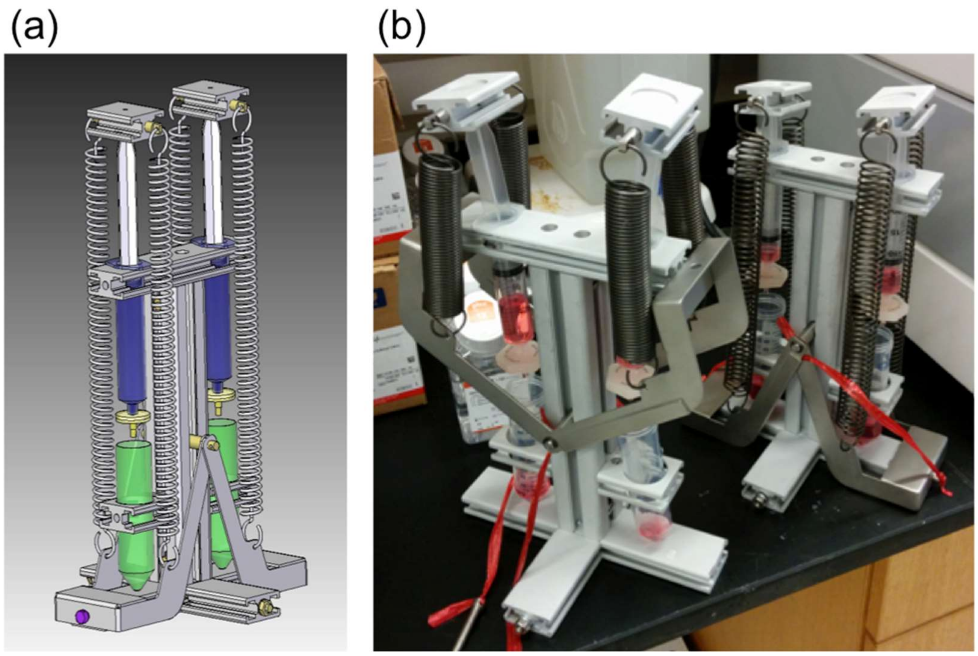

Supplementary Figure 2. A mechanical syringe pump was designed **(a)** and manufactured **(b)** to automate filtration through the 0.02 um pores.

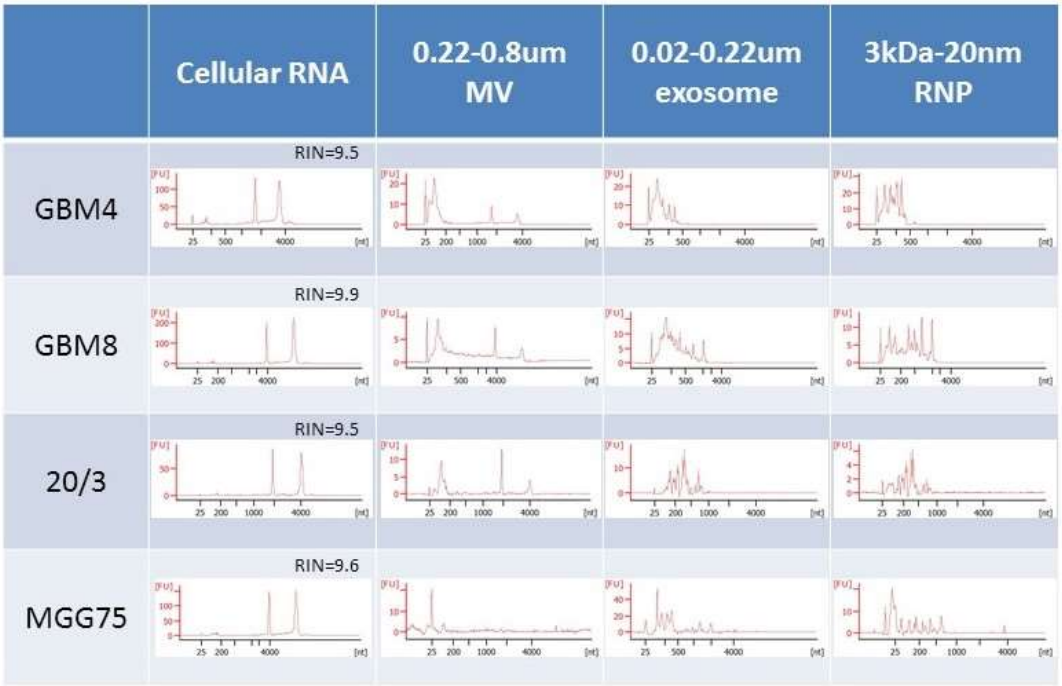

Supplementary Figure 3. RNA profiles of cellular and extracellular RNA have been examined by the Agilent Bioanalyzer 2100.

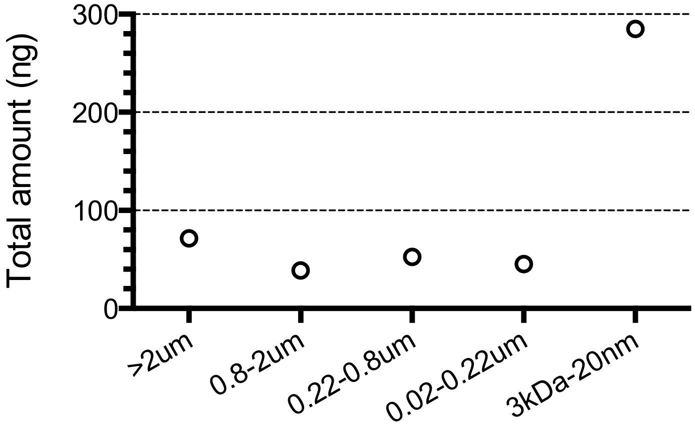

Supplementary Figure 4. RNA yield of complexes isolated from 500mL of the fresh media used for GSC cultures.

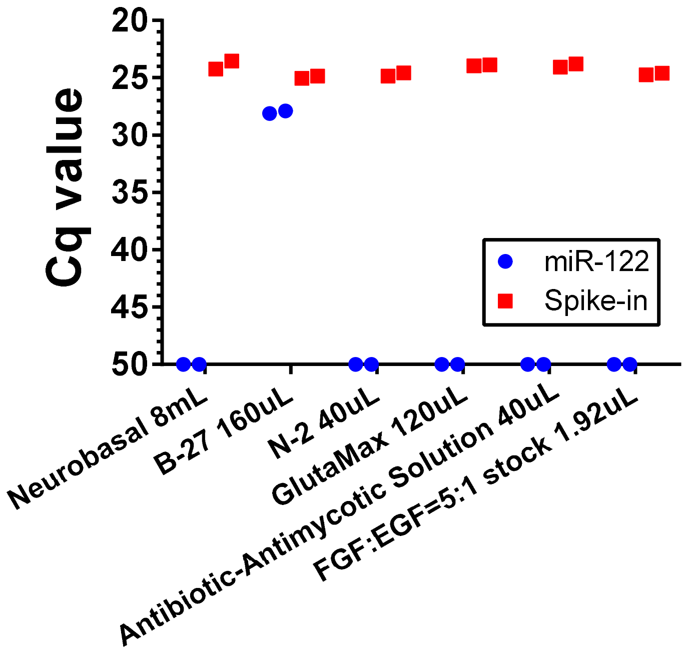

Supplementary Figure 5. miR-122 in fresh media originates from the B-27 supplement. Total RNA was isolated from each component of the fresh 3D media, from relative volumes reflecting their proportions in the complete media. The same volume (5uL from 20uL eluted) of isolated RNA was used as input for reverse transcription. miR-122 was abundant in B-27, but not detected in other components during 50 cycles of qPCR. Spike-in was added during reverse transcription. N=2 PCR, in duplicates.

$$A_{exFrac_i\_CM\_corrected} = \frac{\dfrac{A_{exFrac_i\_CM} \times M_{exFrac_i\_CM}}{V_{CM}} - \dfrac{A_{exFrac_i\_FM} \times M_{exFrac_i\_FM}}{V_{FM}}}{\dfrac{M_{exFrac_i\_CM}}{V_{CM}} - \dfrac{M_{exFrac_i\_FM}}{V_{FM}}}$$

A: abundance (fmol per ug total RNA, normalized by spike-ins)
M: yield (ug of RNA isolated from each fraction)
V: volume (mL of media used for exRNA isolation)

$exFrac_i$ with i=1, 2, 3 correspond to MV, exosome and RNP, respectively.
CM: conditioned media
FM: fresh media

Supplementary Figure 6. GSC-released exRNA levels are corrected to the fresh media RNA according to the above formula.

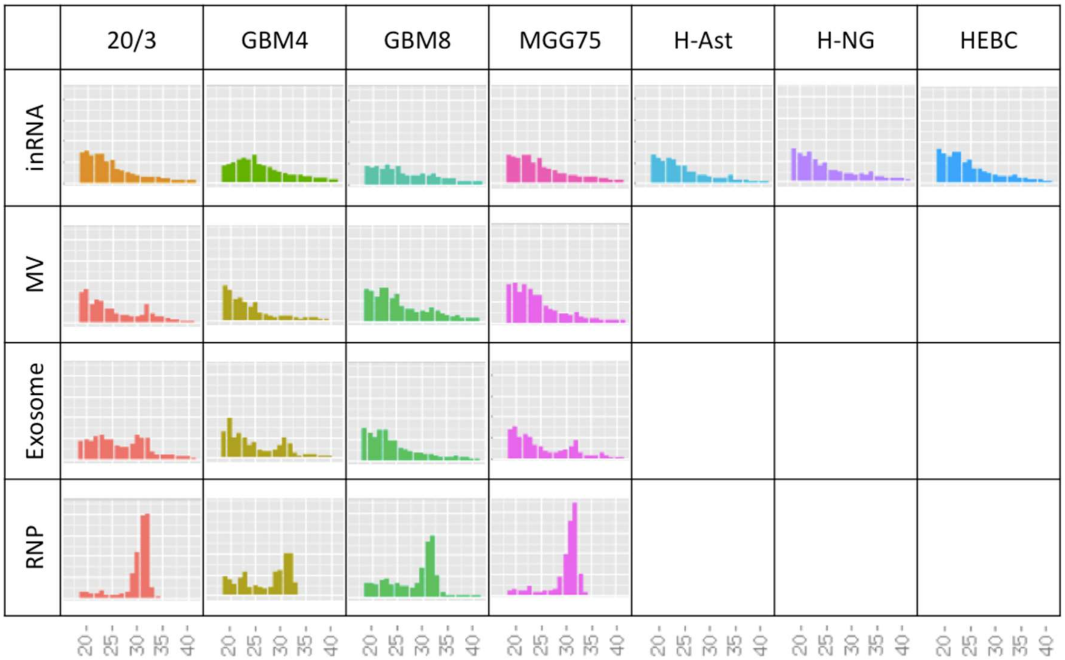

Supplementary Figure 7. The reads' length distribution of the small RNA libraries. The small RNA libraries were not enriched for miRNAs, resulting in the broad distribution of the reads, with no typical miRNA peak observed at 22nt. tRNA and Y RNA fragments are predominant in the RNP fraction, producing the sharp ~32nt peak.

(a)

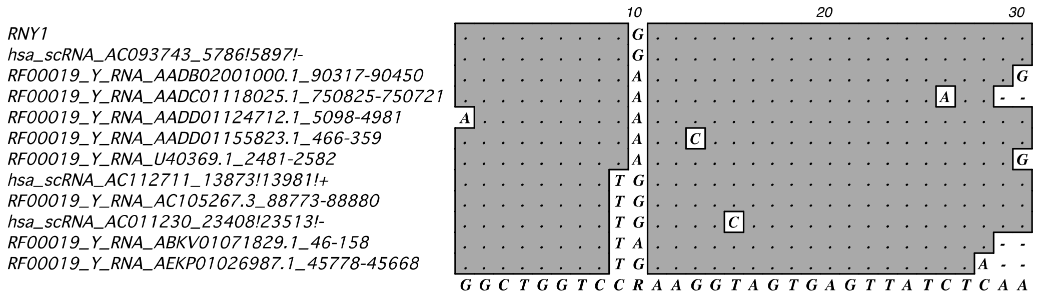

(b)

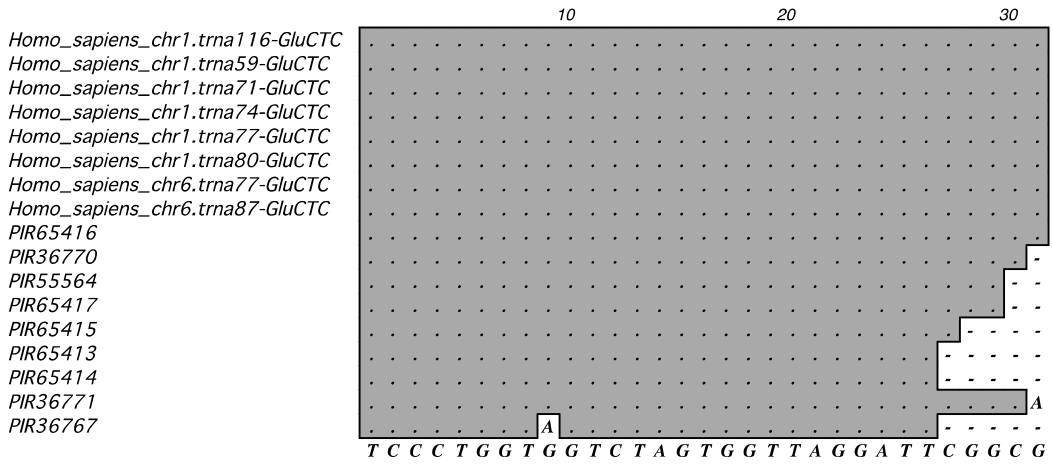

Supplementary Figure 8. Top abundant scRNA and piRNA species are highly similar to Y RNA fragments **(a)** and tRNA fragments **(b)**, respectively.

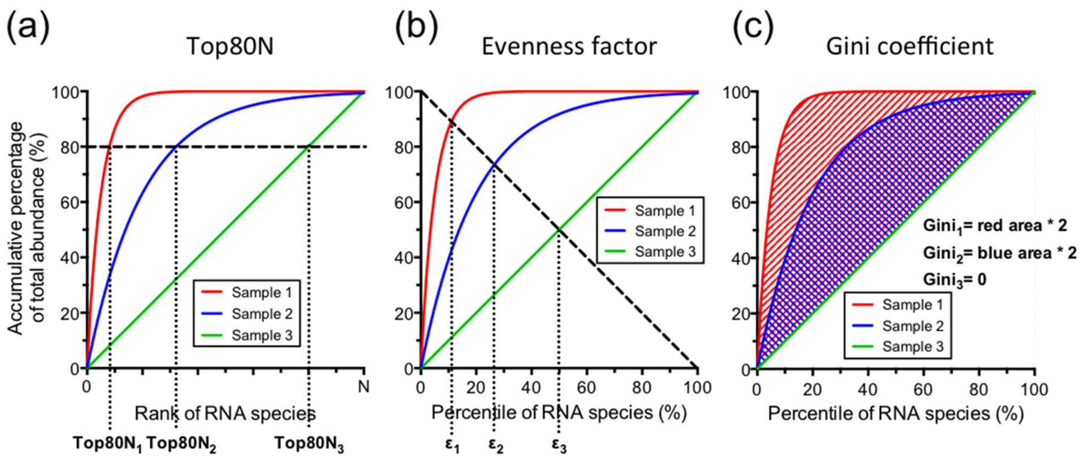

Supplementary Figure 9. Comparison between the traditional evaluation of inequality with pre-set cutoff, and two other evaluation systems, is illustrated by three examples: Sample 1 with unequal RNA species, Sample 2 with moderately unequal RNA, and Sample 3 with equal RNA representation. In the traditional evaluation **(a)**, Top80N can range from 1 to 0.8*N, with N representing the number of RNA species analyzed. Alternative thresholds could be set up. In contrast, Evenness factor (ε) is determined by the intersection between the curve depicting RNA accumulation and the diagonal line with the negative slope **(b)**. Gini coefficient is twice the area between the curve depicting RNA accumulation and the diagonal line with the positive slope **(c)**. For the latter two evaluations, no pre-set threshold is required. Higher Top80N or EvenF factors, but lower Gini coefficient, correspond to lower inequality (or higher equality).

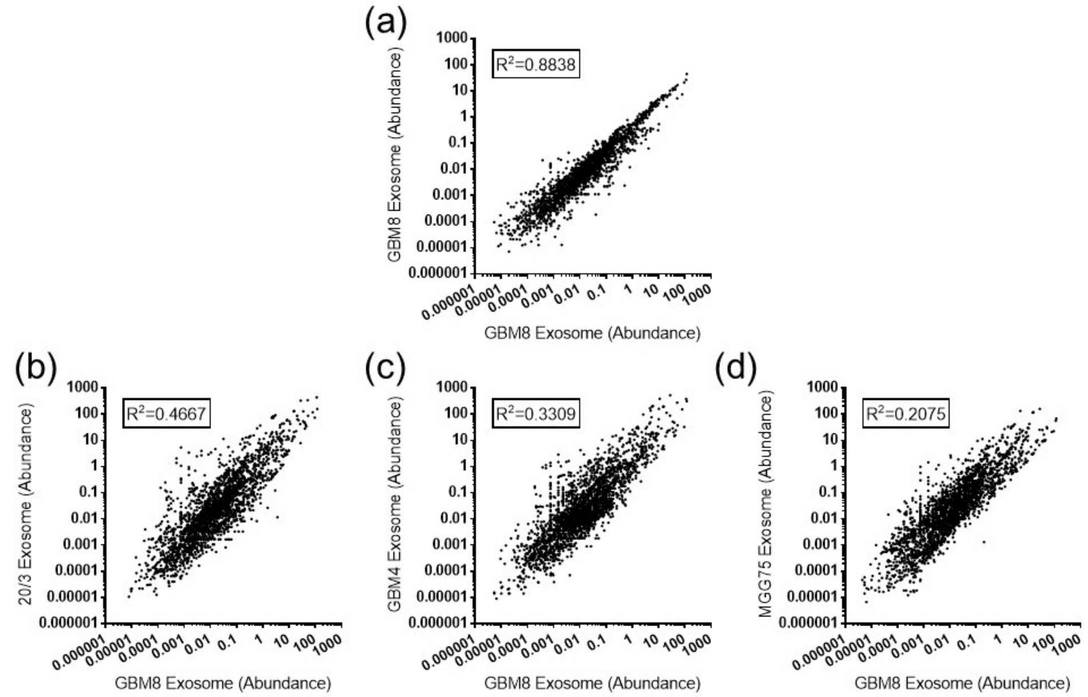

Supplementary Figure 10. Correlation of small RNA composition between two sets of GBM8 exosomes, and exosomes derived from other GSC cultures. **(a)** Two isolations of exRNA from different passages of GBM8 are compared. The y-axis shows the abundance of each RNA species in the RNAseq dataset 1 (culture 1, no rRNA depletion performed). The x-axis shows the abundance of each RNA species in the RNAseq dataset 2 (culture 2, with rRNA depletion). **(b-d)** GBM8 exosomal small RNA composition is compared with 20/3, GBM4, and MGG75, respectively, all analyses performed with rRNA depletion.

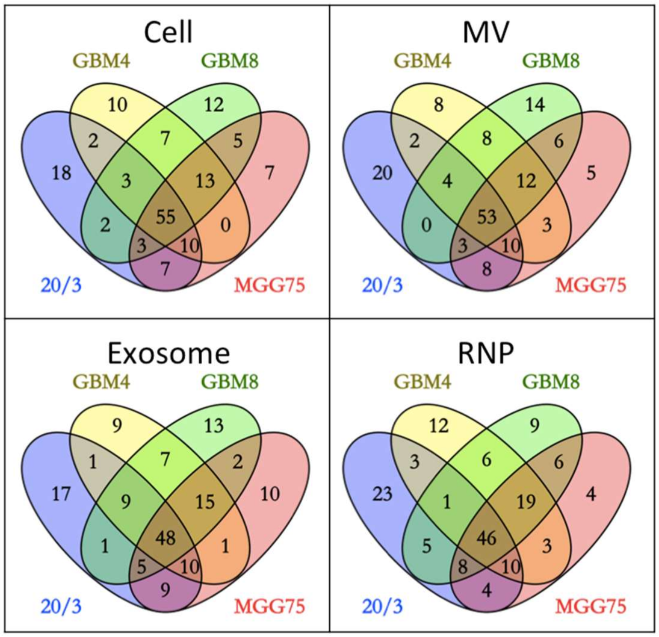

Supplementary Figure 11. Venn diagrams show the numbers of common miRNA species among 100 most abundant miRNAs, in four GSC cultures and their exRNA fractions.

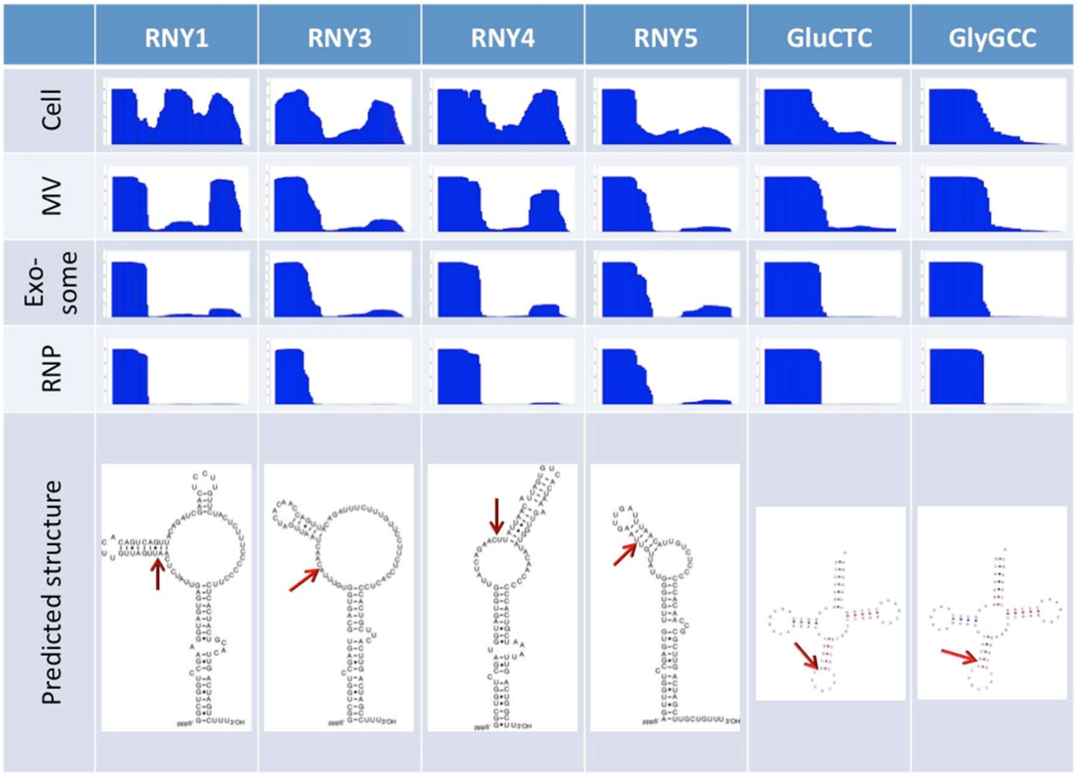

Supplementary Figure 12. Depth plots of the reads' coverage demonstrate that precisely processed fragments of Y RNA and tRNA are found in exosomes and RNPs. Predicted Y RNA and tRNA structures were adopted from O'Brien et al. [1], Copyright (1993) National Academy of Sciences, U.S.A., and GtRNAdb, respectively.

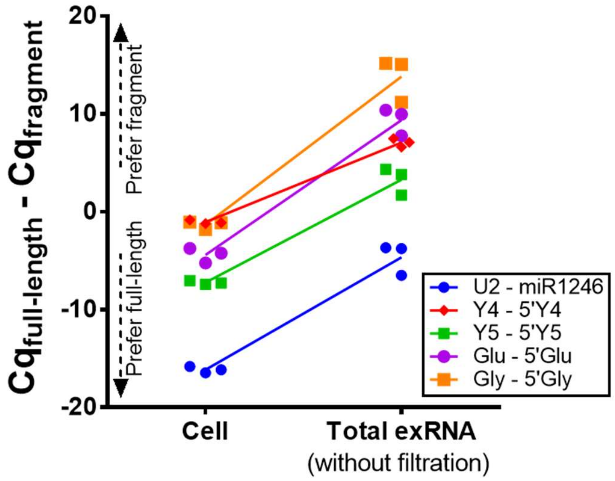

Supplementary Figure 13. RNA fragments are preferred over their full-length parental transcripts in unfractionated exRNA. Upon brief centrifugation at 300 g and 2,000 g, GBM8 conditioned media was directly concentrated at the 3kDa filters and RNA isolated and analyzed by qRT-PCR. N=3 aliquots of GBM8 conditioned media.

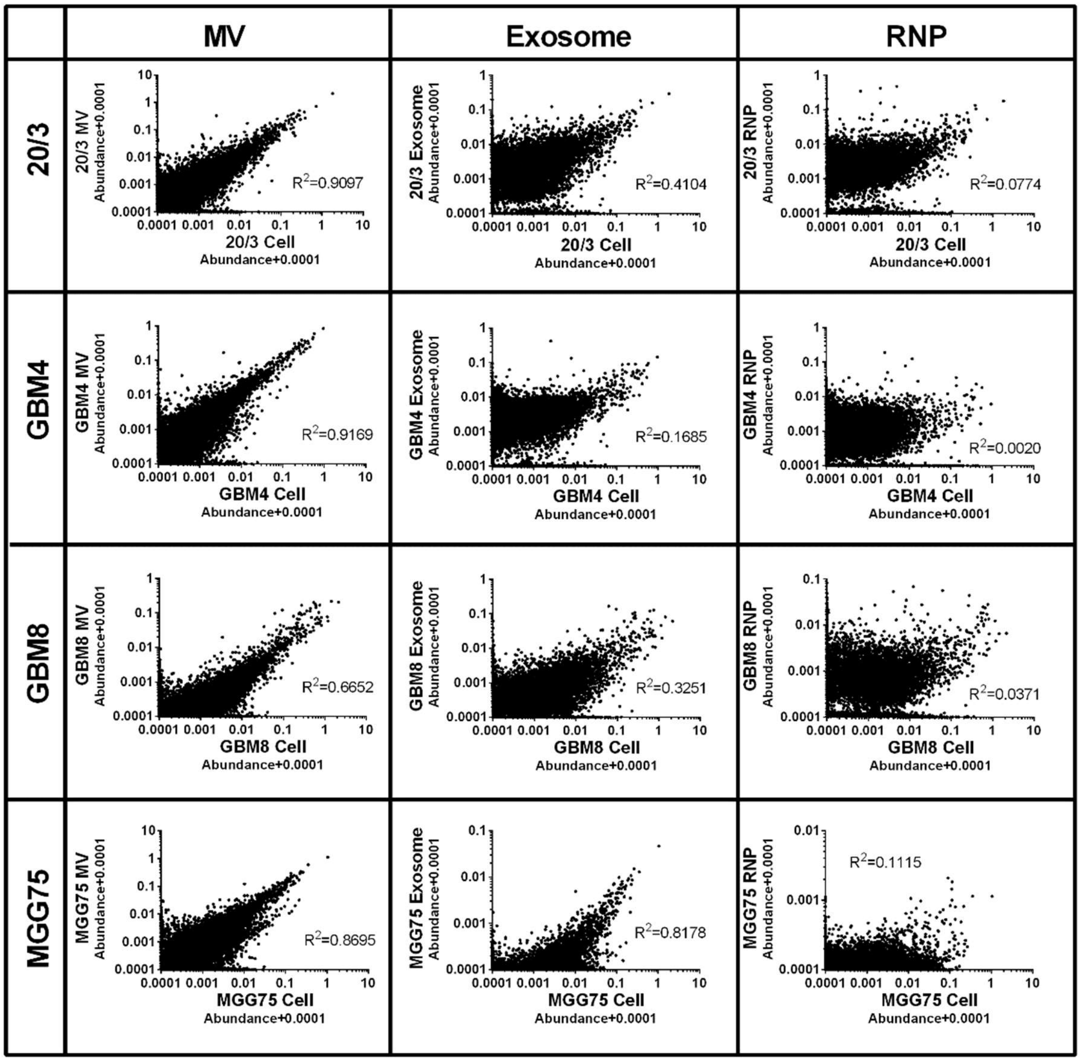

Supplementary Figure 14. Correlation of mRNA abundance between GSC cellular and extracellular fractions.

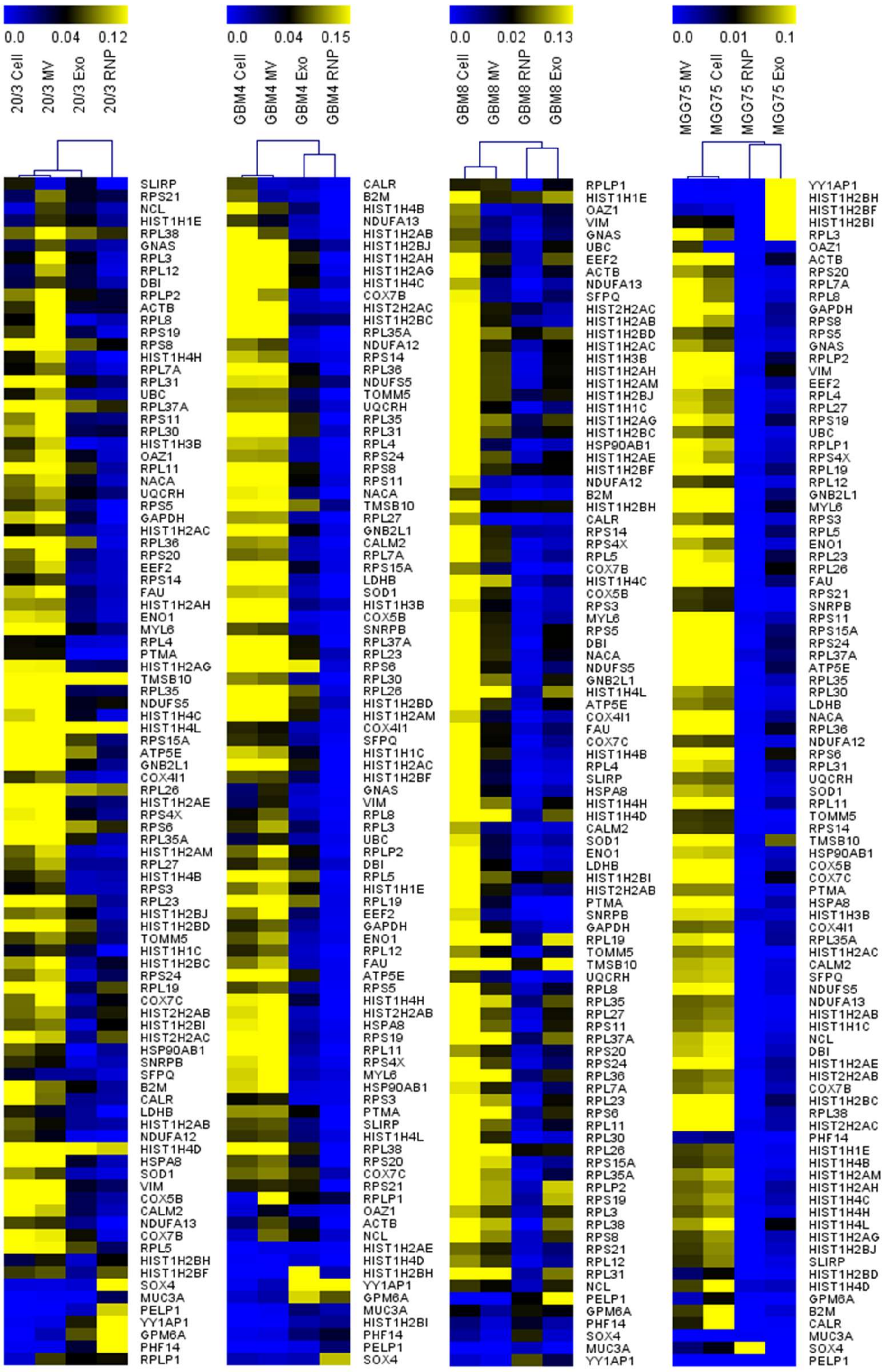

Supplementary Figure 15. Clustering analysis of the most abundant mRNA species is GSC cultures. The scale bars represent the abundance.

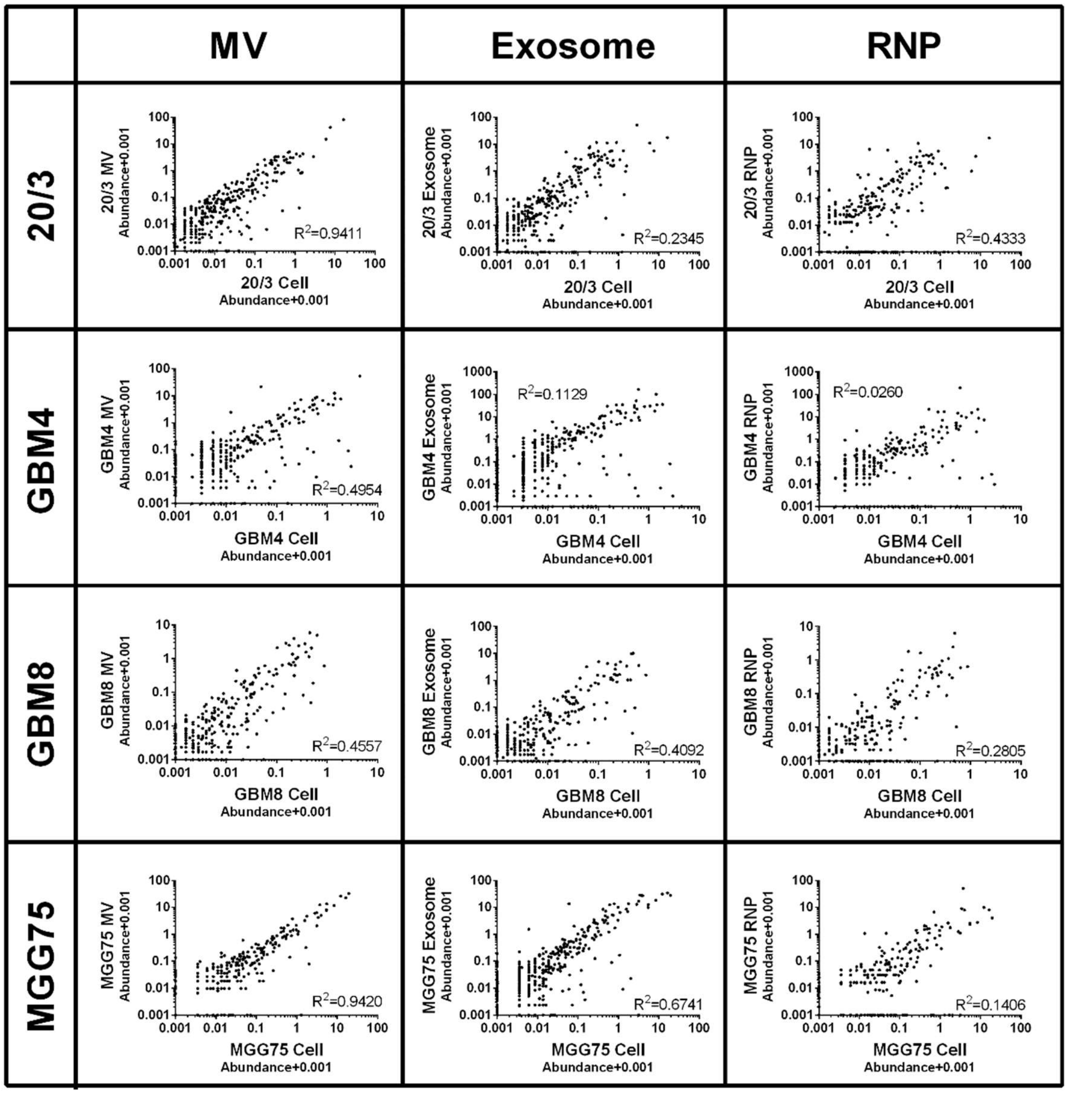

Supplementary Figure 16. Correlation of miRNA abundance between GSC cellular and extracellular fractions.

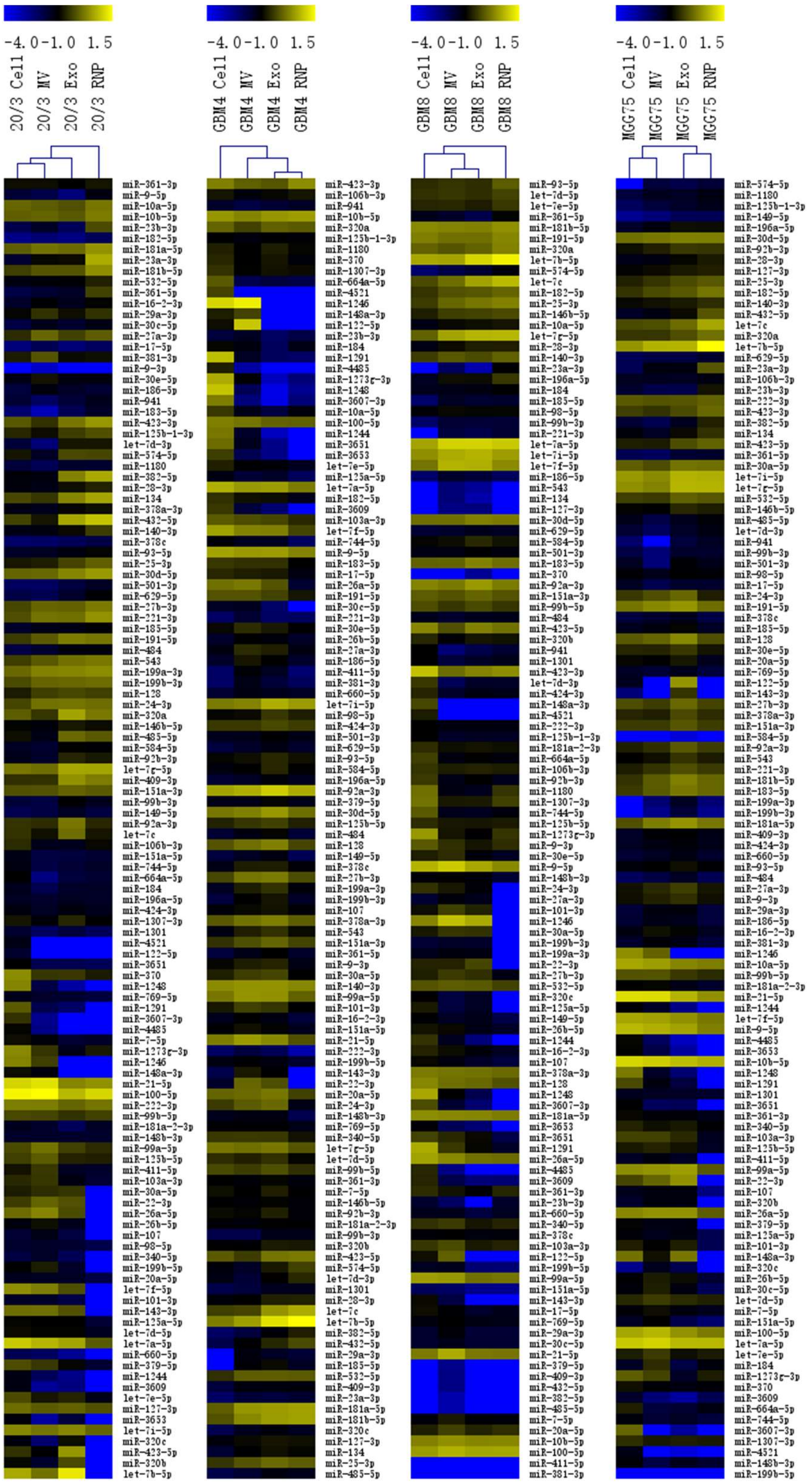

Supplementary Figure 17. Clustering analysis of the most abundant miRNA species is GSC cultures. The scale bars represent the log transformed abundance.

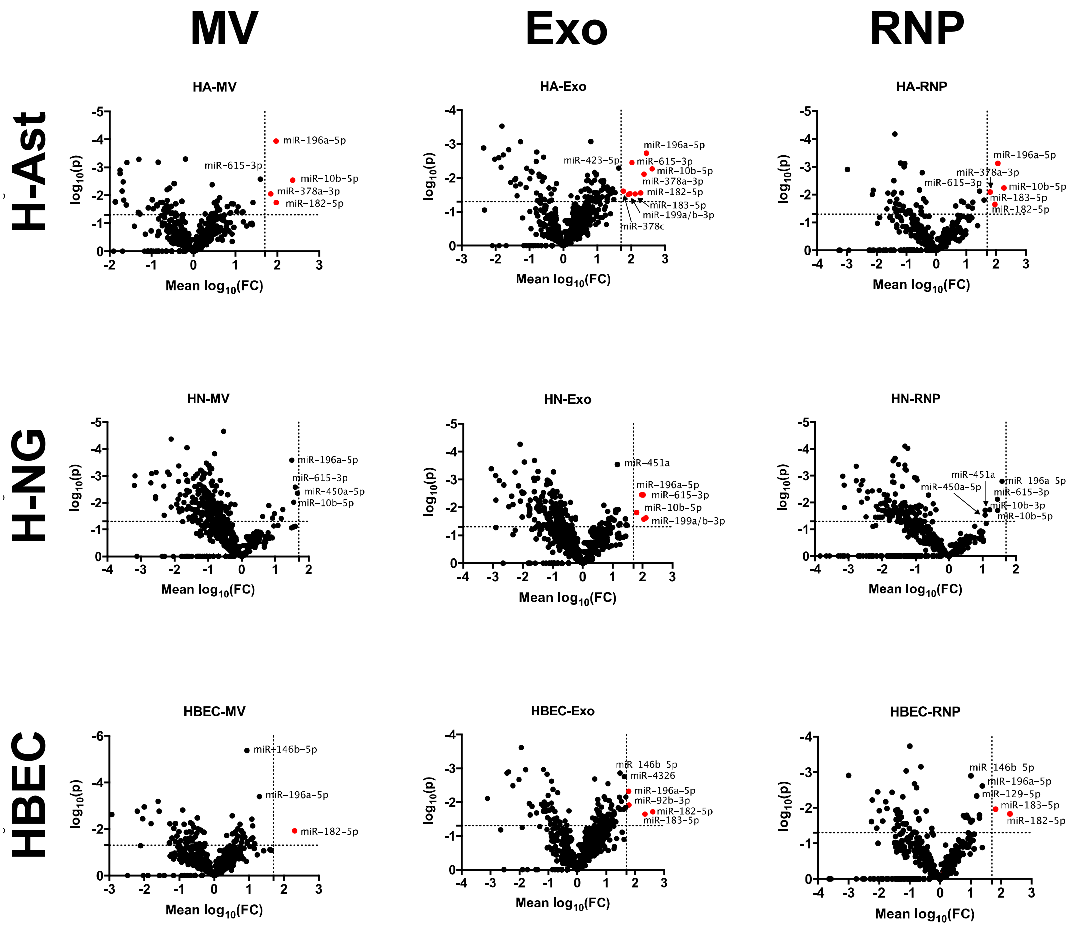

Supplementary Figure 18. Comparative analyses of GSC-released miRNAs and cellular miRNome of the normal cells of the brain, including astrocytes (H-Ast), neuroglia (H-NG) and endothelial cells (HBEC), predict the most impactful GSC miRNAs in tumor-to-microenvironment communication. Extracellular fractions of GSC cultures were compared to primary normal cells, based on the corresponding miRNAseq datasets. The horizontal axis of Volcano plots shows the fold difference, and the vertical axis shows the statistical significance. Putative most impactful miRNAs are colored in red using the following cutoff: $p<0.05$ (t test) and fold change between exGSC/normal cells>50.

(a)

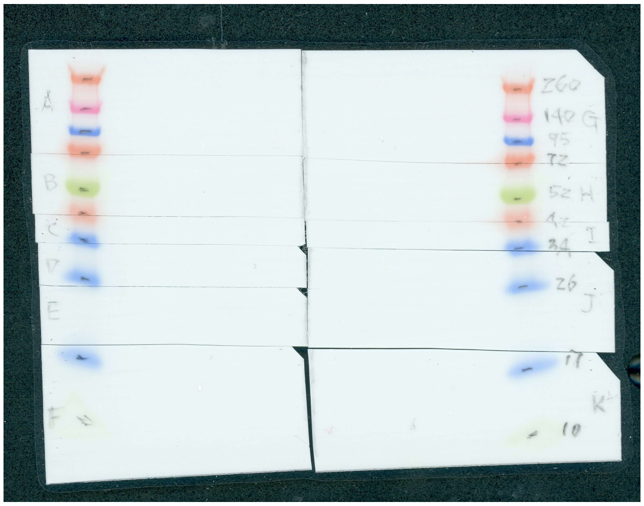

(b)

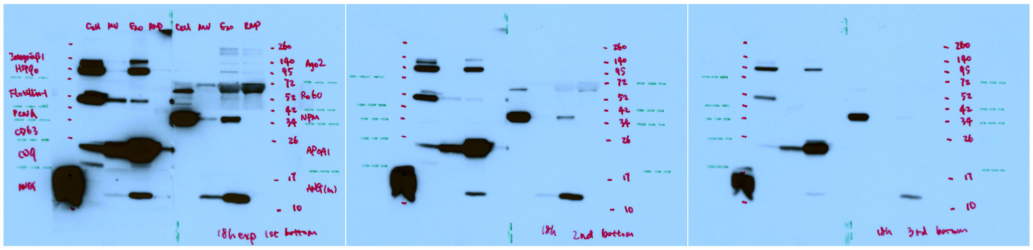

(c)

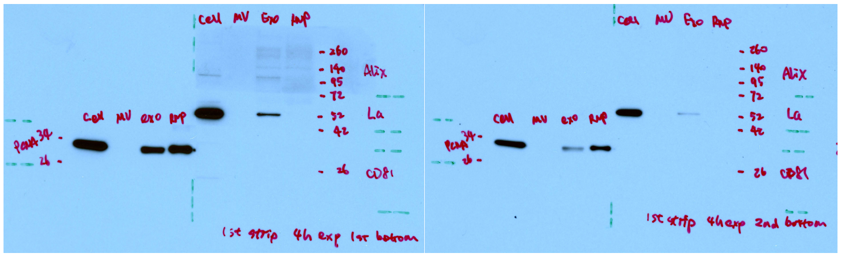

Supplementary Figure 19. Uncropped scans of immunoblots. Due to the limiting amount of the extracellular material available, PVDF membranes were cut to several pieces for blotting with different antibodies **(a)**. Various exposures were used for visualization of different proteins (short to long from right to left) **(b)**. Selected membranes were stripped once, the stripping efficiency was validated, and the membranes reblotted with additional antibodies **(c)**.

Supplementary Table 1. Detected circular RNAs in GSC cellular or extracellular fractions.

	Mean read count in			
	**Cell**	**MV**	**Exosome**	**RNP**
#All circRNA#	418.25	264.5	32.5	12.5
hsa_circ_002173	26.25	5.25	1	0
hsa_circ_0004449	24.25	7	0	0
hsa_circ_000003	21	14	0.75	0
hsa_circ_002057	18	11.75	0	0
hsa_circ_001812	15.75	8.75	0.25	0
hsa_circ_000017	13.5	8.5	0	0
hsa_circ_002121	12.75	8.75	0	0
hsa_circ_001422	8.5	0	0	0
hsa_circ_000374	8	3	0	0
hsa_circ_000193	7.5	0.25	0	0
hsa_circ_002043	6.5	3	0	0
hsa_circ_001251	6.25	1.5	0	0
hsa_circ_000720	5.75	1	0	0
hsa_circ_0001794	5.75	0	0	0
hsa_circ_001317	5.5	1.25	0	0
hsa_circ_000314	5.5	1	0	0
hsa_circ_002176	5.5	0.5	0	0
hsa_circ_002131	5.25	2.5	0	0
hsa_circ_001778	5.25	0	0	0
hsa_circ_001472	5	0.75	0	0
hsa_circ_001502	5	0.5	0	0
hsa_circ_0004207	2.5	8.75	0	0
hsa_circ_0004929	2	5.75	0	0
hsa_circ_0004283	1.75	6.5	0	0
hsa_circ_0001057	0	6.75	0	0
hsa_circ_001494	0	5.25	0	0
hsa_circ_000771	0	2.75	5.25	12
hsa_circ_000778	0	1.75	6.75	0
hsa_circ_0002244	0	0	8.25	0

circRNAs with mean read count less than 5 in all four fractions were excluded.

Supplementary Table 2. Evaluation of inequality of cellular and extracellular RNA by three independent measures

RNA category	Measure of inequality	Cell	MV	Exosome	RNP
All long RNA	EvenF	6.37±1.49	4.71±1.53	4.67±1.04	4.92±0.47
	Top80N	1040.0±899.4	754.8±751.8	176.3±173.3	58.0±55.3
	Gini	0.971±0.014	0.979±0.012	0.983±0.008	0.984±0.002
--mRNA	EvenF	11.70±0.89	12.12±0.36	14.27±3.85	16.30±2.45
	Top80N	2458.5±499.8	2611.5±225.9	4534.0±2074.9	5403.8±1309.3
	Gini	0.917±0.013	0.912±0.006	0.863±0.063	0.834±0.037
--ncRNA	EvenF	2.59±0.72	4.26±0.89	2.89±1.76	3.81±0.40
	Top80N	36.5±27.5	44.0±23.4	7.3±5.0	4.8±3.1
	Gini	0.994±0.003	0.985±0.005	0.988±0.011	0.99±0.002
All small RNA	EvenF	3.94±0.26	3.12±0.10*	2.26±0.19**	0.91±0.15***
	Top80N	169.0±29.0	87.8±7.1*	61.5±9.8*	14.0±1.9**
	Gini	0.986±0.002	0.991±0.001*	0.994±0.001**	0.999±0.000***
--miRNA	EvenF	6.09±0.34	5.27±0.12	5.39±0.35	4.76±0.30*
	Top80N	23.3±2.8	19.5±0.9	23.0±1.9	18.5±3.1
	Gini	0.966±0.004	0.973±0.001	0.968±0.002	0.976±0.003
--snoRNA	EvenF	16.62±1.79	17.98±0.72	16.24±1.27	9.41±2.57
	Top80N	50.8±14.5	63.3±5.0	52.8±8.1	19.5±9.4
	Gini	0.840±0.029	0.814±0.012	0.835±0.022	0.925±0.025
--snRNA	EvenF	11.63±0.57	8.10±0.69**	6.98±0.58**	5.99±0.99**
	Top80N	43.0±4.7	22.8±4.9*	21.3±2.7**	16.3±5.7*
	Gini	0.905±0.008	0.944±0.009*	0.955±0.005**	0.964±0.010**
--SRP RNA	EvenF	17.81±0.79	15.57±0.45*	12.32±1.05**	14.19±1.43
	Top80N	83.0±9.1	58.5±3.3*	40.3±5.0**	51.0±11.4
	Gini	0.788±0.016	0.828±0.008	0.876±0.017**	0.843±0.022
--tRNA	EvenF	19.20±0.45	17.41±0.45*	11.61±0.89***	8.48±0.45***
	Top80N	10.3±0.5	8.3±0.5*	4.3±0.5***	2.8±0.3***
	Gini	0.761±0.009	0.804±0.005**	0.896±0.012***	0.928±0.006***
--vRNA	EvenF	31.25±6.25	31.25±6.25	25.00±0.00	18.75±6.25
	Top80N	1.8±0.5	1.5±0.3	2.0±0.0	1.3±0.3
	Gini	0.476±0.082	0.492±0.069	0.416±0.007	0.608±0.064
--Y RNA	EvenF	11.27±0.98	8.33±0.56*	6.54±0.27**	5.88±0.38**
	Top80N	8.3±1.7	7.0±0.6	6.5±1.0	5.3±0.8
	Gini	0.894±0.011	0.919±0.005	0.944±0.006**	0.953±0.005**

EvenF: EvenF factor; Gini: Gini coefficient; Top80N: the number of species accounting for 80% of total RNA in the indicated class of transcripts. Significant differences with the cellular RNA composition are labeled by asterisks: *, p<0.05; **, p<0.01; ***, p<0.001; t test. Data shown as mean±s.e.m. Significantly increased inequality was marked in red.

Supplementary Table 3. Primer sequences for the long RNA RT-PCR analysis

mRNA	PCR primer-F	PCR primer-R	Annealing temperature	Extension time
RPS27	GACCTACGCACACGAGAACAT	TGGTTTCCCACTCATCTTGACT	67.5 ℃	12 s
RPLP2	TTCCTTTTCCTCCCTGTCGCC	AGGCTTTATTTGCAGGGGAGC	70.5 ℃	12 s
RPS6	GCGTTCAGCTGCTTCAAGATG	AGGAAAGTCTGCGTCTCTTCG	68.0 ℃	12 s
EEF2	ACTTCACGGTAGACCAGAT	GACTTCCTGCCTGCTAGA	56.5 ℃	45 s
YY1AP1	GAAGCAAATGAGCTGATGG	GGGTTTCCTATTGGTTACAC	58.0 ℃	45 s
PROM1	GAGTGAATGACCCCCTTGGAG	GTTTCATCCATGCTGGACACC	70.0 ℃	67 s

Supplementary Table 4. Primer sequences for the mRNA qRT-PCR analysis

**mRNA**	**Species**	**PCR primer-F**	**PCR primer-R**
COL1A2	Human	CCGTGGCAGTGATGGAAGTGT	ACCAGCAGGACCAGCGTTAC
PTEN	Human	GCAGAAAGACTTGAAGGCGTA	TTGGCGGTGTCATAATGTCT
EEF2	Human	CTGGACAGCGAGGACAAGGA	GAGGGCAGGTGGATGGTGAT
RPS6	Human	AAGTTGCTGCTGACGCTCTG	TTCTCCAGTTCTCCTTGGTCTGT
Actb	Mouse	GGCACCACACCTTCTACAATG	GGTACGACCAGAGGCATACA
Timp3	Mouse	ACATTCACACGGAAGCCTCTG	CCAGGTGGTAGCGGTAATTGAG
Rassf9	Mouse	ATGGCTCCCTTTGGAAGAAAC	GGCTTCGTGTTCCTCAAGTAG
Ptgfr	Mouse	GTGATCCAGTGCTCAGAAGTG	GCAGACATCTCGGCATCCT
Hmgb3	Mouse	CTTATGTCACCAAGGCAGCAA	CTCCTCCTCTTCCTCTTCTTCC
Hapln1	Mouse	TGCTTCTCCTGGTGCTGATT	CCTTGGCTTGTTCTGCTTCC
Cep110	Mouse	TGGCGAATGGTCAAGAGGAG	CTGAAGCTGAAGGTCTGTAAGG

Supplementary References

1 O'Brien, C. A., Margelot, K. & Wolin, S. L. Xenopus Ro ribonucleoproteins: members of an evolutionarily conserved class of cytoplasmic ribonucleoproteins. *Proceedings of the National Academy of Sciences of the United States of America* **90**, 7250-7254 (1993).
