# Supplementary material for: Coding and noncoding landscape of extracellular RNA released by human glioma stem cells
Source: Nat Commun. 2017 Oct 26;8:1145. doi: 10.1038/s41467-017-01196-x (PMC5658400; doi:10.1038/s41467-017-01196-x)
Supplement: Supplementary file 3 — Description of Additional Supplementary Files [file 41467_2017_1196_MOESM3_ESM.pdf]

Description of Additional Supplementary Files

File Name: Supplementary Data 1
Description: Characteristics of the common, most abundant GSC mRNA species.

File Name: Supplementary Data 2
Description: RNA species enriched in extracellular fractions. RNA species with mean of log10-transformed fold change more than 1.0, and p value (t test) less than 0.05 are listed.

File Name: Supplementary Data 3
Description: Coding-free tool to calculate Chi^2

File Name: Supplementary Data 4
Description: Coding-free tool to calculate inequality
